# Identification and evaluation of antiviral activity of novel compounds targeting SARS-CoV-2 virus by enzymatic and antiviral assays, and computational analysis

**DOI:** 10.1080/14756366.2024.2301772

**Published:** 2024-01-14

**Authors:** Ivana Nemčovičová, Katarína Lopušná, Iveta Štibrániová, Fabio Benedetti, Federico Berti, Fulvia Felluga, Sara Drioli, Mattia Vidali, Jaroslav Katrlík, Lucia Pažitná, Alena Holazová, Jana Blahutová, Simona Lenhartová, Monika Sláviková, Boris Klempa, Miroslav Ondrejovič, Daniela Chmelová, Barbora Legerská, Stanislav Miertuš, Mária Klacsová, Daniela Uhríková, Lukáš Kerti, Vladimír Frecer

**Affiliations:** a Biomedical Research Center, Institute of Virology, Slovak Academy of Sciences, Bratislava, Slovakia; b Department of Chemical and Pharmaceutical Sciences, University of Trieste, Trieste, Italy; c Institute of Chemistry, Slovak Academy of Sciences, Bratislava, Slovakia; d Department of Biotechnology, Faculty of Natural Sciences, University of Ss. Cyril and Methodius in Trnava, Trnava, Slovakia; e ICARST n.o, Bratislava, Slovakia; f Department of Physical Chemistry of Drugs, Faculty of Pharmacy, Comenius University Bratislava, Bratislava, Slovakia

**Keywords:** SARS-CoV-2 viral cysteine proteases Mpro and PLpro, bis(benzylidene)cyclohexanone inhibitors, *in vitro* antiviral effect, enzyme inhibition assays

## Abstract

The viral genome of the SARS-CoV-2 coronavirus, the aetiologic agent of COVID-19, encodes structural, non-structural, and accessory proteins. Most of these components undergo rapid genetic variations, though to a lesser extent the essential viral proteases. Consequently, the protease and/or deubiquitinase activities of the cysteine proteases M^pro^ and PL^pro^ became attractive targets for the design of antiviral agents. Here, we develop and evaluate new bis(benzylidene)cyclohexanones (BBC) and identify potential antiviral compounds. Three compounds were found to be effective in reducing the SARS-CoV-2 load, with EC_50_ values in the low micromolar concentration range. However, these compounds also exhibited inhibitory activity IC_50_ against PL^pro^ at approximately 10-fold higher micromolar concentrations. Although originally developed as PL^pro^ inhibitors, the comparison between IC_50_ and EC_50_ of BBC indicates that the mechanism of their *in vitro* antiviral activity is probably not directly related to inhibition of viral cysteine proteases. In conclusion, our study has identified new potential noncytotoxic antiviral compounds suitable for *in vivo* testing and further improvement.

## GRAPHICAL ABSTRACT

**Figure:**
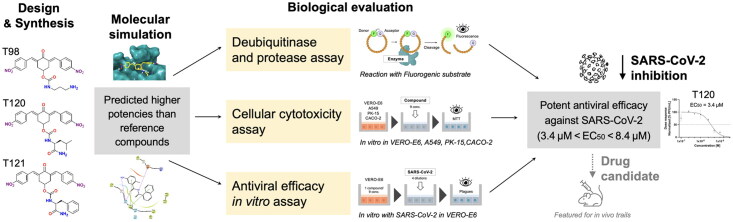

## Introduction

### Coronavirus outbreaks

Several members of the *Coronaviridae* family constantly circulate in the human population and usually cause mild respiratory disease^1^. In contrast, the severe acute respiratory syndrome coronavirus (SARS-CoV) and the Middle East respiratory syndrome coronavirus (MERS-CoV) are transmitted from animals to humans and cause acute respiratory diseases in infected individuals^2^. The coronavirus disease COVID-19 started to spread among humans in December 2019. Since its first outbreak in China, it quickly became a global pandemic. The outbreak of COVID-19 showed us how inadequately prepared we are for diseases caused by emerging (e.g., zoonotic) viruses. Vaccines represent the best strategy to suppress the COVID-19 pandemic. However, their waning protection and a large pool of unvaccinated individuals provide the ground for the spread of the virus and the generation of novel variants, which represent a constant threat for vulnerable people with inadequate immune responses. For those who become infected, more efficient treatment will need to be developed.

Viral cycles are highly dependent on cellular factors and cellular metabolic and signalling pathways; therefore, the number of possible antiviral drug targets is limited. However, almost all viruses encode unique proteins and enzymes that can serve as specific targets for antiviral therapy. One of the main goals of modern drug development efforts is to design compounds that specifically inhibit viral targets or cellular targets essential for virus replication. To complement the weaponry available to combat the SARS-CoV-2 virus, here we focus on the development and evaluation of new small-molecule inhibitors targeted against viral enzymes.

### Variants, vaccines, and antivirals interconnected with genome stability

Genetic diversification of SARS-CoV-2 was initially considered slow when the virus spread in 2019 and early 2020. The first official variant, a single spike D614G mutation found in early European lineages, was linked to more efficient transmission^3^ and rapidly spread to become the dominant viral strain worldwide. Later in 2020, multiple variants emerged that launched regional epidemics. According to the WHO (World Health Organisation), five main variants have been identified (Alpha, Beta, Gamma, Delta, and Omicron). All have characteristic mutations closely described at: www.ecdc.europa.eu/en/covid-19/variants-concern. The spike glycoprotein appears especially prone to accumulate mutations^4^ and all circulating variants have some mutations that favour evasion from the host immune response^5^. However, sera studies and emerging real-world evidence indicate that Omicron escapes adaptive immunity initiated by previous infection or vaccination^6^. Therefore, it is important to develop antiviral agents acting directly on SARS-CoV-2 as an alternative tool in the fight against the virus. Several antiviral therapeutics have been approved or are advancing in clinical development^7^. As an example of the direct-acting small-molecule SARS-CoV-2 antivirals that have received approval or emergency use authorisation do not bind to the variable spike protein, but target conserved viral RNA-dependent RNA polymerase or conserved viral cysteine proteases (M^pro^ or PL^pro^). Remdesivir, a mono-phosphoramidate prodrug of the nucleoside GS-441524, originally developed to treat Ebola virus infections, inhibits the RNA polymerase of SARS-CoV-2. It was the first antiviral approved or authorised for emergency use to treat COVID-19 in several countries. Additionally, molnupiravir (MK-4482 or EIDD-2801), a small molecule ribonucleoside prodrug of *N*-hydroxycytidine, originally developed against different RNA viruses such as influenza^8^, has recently received an emergency use authorisation for the treatment of mild-to-moderate COVID-19. Another approved small-molecule antiviral chemotherapeutic drug, paxlovid (nirmatrelvir/ritonavir), irreversibly inhibits viral protease M^pro^ and stops viral replication of SARS-CoV-2^9^.

### Proteases play an essential role in the replication of SARS-CoV-2 and are genetically stable

The SARS-CoV-2 viral genome encodes several structural proteins (e.g., capsid spike glycoprotein), non-structural proteins (e.g., 3-chymotrypsin-like or main protease abbreviated as 3CL^pro^ or M^pro^, papain-like protease PL^pro^, helicase, and RNA-dependent RNA polymerase) and accessory proteins. Some of these components undergo genetic variations^10^, while mutations within cysteine proteases M^pro^ and PL^pro^ were also found and recently also documented^11^. The viral M^pro^ cleaves the two polyproteins (pp1a and pp1ab) of SARS-CoV-2 at multiple locations, resulting in various non-structural proteins, which are key for viral replication^12^. PL^pro^ is similarly involved in viral replication, but it also plays a role in altering the host antiviral immune response^13^. Both enzymes were validated as potential antiviral drug targets^14^^,^^15^ and their higher genomic stability in all variants of SARS-CoV-2 makes them attractive in this respect. For example, nirmatrelvir (PF-07321332), is an oral-available M^pro^ inhibitor. When treatment is started during the first days after the appearance of symptoms, it results in approximately 90% protection against severe COVID-19 and hospitalisation^9^. Unfortunately, nirmatrelvir – the active component of paxlovid – is rapidly metabolised in the liver. Therefore, ritonavir is co-administered with nirmatrelvir to dampen metabolic conversion by cytochrome CYP3A, which can cause adverse effects in polymorbid patients^16^^,^^17^. Moreover, the construction and analysis of several recombinant SARS-CoV-2 clones showed that the main protease mutations mediated only low-level resistance to nirmatrelvir, whereas greater resistance required accumulation of additional mutations^18^. Although, these findings indicate that SARS-CoV-2 resistance to nirmatrelvir does readily arise via multiple pathways *in vitro*, the specific mutations form a foundation to study the mechanism of resistance in more detail^18^. Although the M^pro^ and PL^pro^ genes can be affected by evolutionary mutations^18^^,^^19^, the viability of the SARS-CoV-2 does not appear to be compromised^20^^,^^21^.

### Deubiquitination, an important function of the SARS-CoV-2 machinery

In addition to polyprotein processing activity, SARS-CoV-2 PL^pro^ possesses a characteristic deubiquitinating activity (DUB) (including the deconjugation of other ubiquitin-like modifiers)^21–23^, as was already observed for analogous SARS-CoV PL^pro^^24^^,^^25^ and for an adenoviral protease^24^^,^^26^. This specific activity interferes with host cell processes and contributes to the ability of the SARS-CoV-2 virus to evade cell defence mechanisms^13^. The deubiquitination function would greatly impact the value of PL^pro^ as a therapeutic target and provide a framework for the development of antivirals to treat SARS-CoV-2^27–29^. Strategies for the design of PL^pro^ inhibitors must also consider the potentially overlapping specificity of this protease with those of cellular deubiquitinating enzymes.

### Bis(benzylidene)cyclohexanones (BBC) are potent deubiquitinase inhibitors

**Figure 1.**
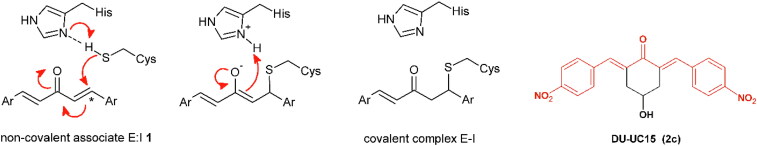
**Left.** Reaction of the 1,5-diaryl-3-oxo-1,4-pentadienyl pharmacophore with thiol group at the catalytic site (showing Cys111 and His272) of SARS-CoV-2 PL^pro^. Initially, a non-covalent enzyme-inhibitor association is formed (E:I, **1**) leading to a covalent adduct. **Right.** Structure of BBC DU-UC15 (2c) with the 1,5-diaryl-3-oxo-1,4-pentadienyl pharmacophore in red.

Dienones are synthetic analogues of curcumin known for their antineoplastic, anti-inflammatory, antiviral, and antiparasitic properties^30^. These varied biological activities are due to the ability of the 1,5-diaryl-3-oxo-1,4-pentadienyl pharmacophore to covalently target biological thiols, including the catalytic cysteines present in many enzymes (Figure 1)^31^.

Recently, the Trieste University group has described the development of new antineoplastic agents in which the 1,5-diaryl-3-oxo-1,4-pentadienyl pharmacophore is embedded in the 4-hydroxycyclohexanone scaffold^32^. BBC DU-UC15 (formerly: **2c**) is a potent inhibitor of several cysteine-dependent deubiquitinases, preventing hydrolytic cleavage of the bond between ubiquitin and target proteins. This, in turn, affects the degradation of damaged proteins by the ubiquitin proteasome system, causing proteotoxic stress and apoptosis in tumour cells. Accordingly, the DU-UC15 inhibitor shows antiproliferative activity in several models, while modified inhibitors, optimised for delivery, show anticancer activity *in vivo*^32–35^.

As both proteases encoded by SARS-CoV-2 are cysteine dependent, we foresee the possibility that the compound DU-UC15 and its congeners might covalently inhibit these enzymes with the same mechanism shown in Figure 1. Substitutions in compound DU-UC15 were introduced to furnish longer chains ending with functional groups capable of reaching the catalytic centres of protease or deubiquitinase of PL^pro^ through relatively narrow substrate channels. Such chains contain amino acids and short peptides or cationic end groups to enhance the interactions with the deubiquitinase site. In addition, inhibition of the specific deubiquitinase activity of PL^pro^ would strengthen cellular defences against infection and provide a new approach to SARS-CoV-2 therapy.

### Protease inhibition and antiviral effect – Searching for new scaffolds

The viral proteases M^pro^ and PL^pro^ play an essential role in coronavirus replication by digesting viral polyproteins at many sites; thus, they appear as high-profile targets for antiviral drug discovery^36–40^. The discovery of S-217622 (also called the Shionogi’s ensitrelvir), the first oral non-covalent, non-peptidic SARS-CoV-2 M^pro^ inhibitor as a clinical candidate provided new opportunity in SARS-CoV-2 antiviral development^41^, while covalent inhibitors^42^^,^^43^ also show great promise for the development of new therapeutics for SARS-CoV-2 infection.

Here, we investigate the inhibitory activity of compound DU-UC15 and several other BBCs against both viral proteases. Some known inhibitors of cysteine proteases and proteasome-associated deubiquitinating enzymes have also been included in the study. PL^pro^ inhibition of both protease (PA) and deubiquitinase (DUA) activities was studied using kits from BPS Bioscience Inc. (San Diego, CA, USA). The *in vitro* antiviral activity of the studied compounds and reference inhibitors was also evaluated in SARS-CoV-2 infected cell cultures.

Overall, our study provides new compounds with potential antiviral activity against SARS-CoV-2 with expected mechanism of action related to inhibition of M^pro^ and PL^pro^ protease activity and PL^pro^ deubiquitinase activity. Some of the compounds identified here represent efficient SARS-CoV-2 inhibitors that can impede SARS-CoV-2 replication *in vitro.*

## Materials and methods

### Cell lines

The cell line VERO-E6 (ECACC 85020206, ATCC-CRL-1586) isolated from the kidney of an African Green Monkey, human colon adenocarcinoma cells Caco-2 (ATCC-HTB-37), porcine kidney cells PK-15 (DSMZ-ACC640), and human lung adenocarcinoma cells A549 (DSMZ-ACC107) are mammalian epithelial cell lines used in a variety of biomedical research, nowadays are also the most widely used to replicate and isolate the SARS-CoV-2 virus. The selected cell models are also commonly used in drug tests and were obtained from the Deutsche Sammlung von Mikroorganismen und Zellkulturen GmbH (DSMZ, Braunschweig, Heidelberg, Germany) or from the American Type Culture Collection (ATCC, LGC Promochem GmbH, Wesel, Germany). VERO-E6, PK-15, and A549 cells were cultured in Dulbecco’s modified Eagle medium (DMEM) supplemented with 5% heat-inactivated foetal bovine serum (FBS) and L-glutamine (2 mM). Caco-2 cells were cultured in Eagle’s Minimum Essential Medium (EMEM) supplemented with 10% heat-inactivated foetal bovine serum (FBS) and L-glutamine (2 mM). The cells were kept in a humidified incubator at 37° C and 5% CO_2_. Cells were subcultured regularly 2–3 times a week, keeping them in the exponential growth phase. All cell lines are regularly tested by PCR for common species of mycoplasma by using primers: *5′-ACACCATGGGAGYTGGTAAT-3′* (forward), *5′-CTTWTCGACTTYCAGACCCAAGGCAT-3′* (reverse); and nested PCR primers: *5′-GTGSGGMTGGATCACCTCCT-3′* (forward), *5′-GCATCCACCAWAWACYCTT-3′* (reverse)^44^^,^^45^.

### Preparation of inhibitors

Commercial reagents and solvents were purchased from Sigma-Aldrich. Column flash chromatography was performed on silica gel 60 (230,400 mesh). Reactions were monitored by thin layer chromatography on silica gel plates using UV light as visualising and KMnO_4_ as developing agent. NMR spectra were recorded on a Varian 500 MHz spectrometer at 500 MHz (^1^H) and 125 MHz (^13^C). Chemical shifts are given in ppm (δ). The coupling constants *J* are given in Hertz. NMR ^1^H and ^13^C resonances were assigned using a combination of DEPT, COSY, and HSQC spectra. Electrospray mass spectra were obtained on a Bruker Daltonics Esquire 4000 spectrometer. Yields refer to spectroscopically homogeneous materials (^1^H NMR).

The structures of the inhibitors related to DU-UC15 (**T97**), which are discussed in this study, are reported in Table 1.

**Table 1.** Bis(benzylidene)cyclohexanones studied in this work.

Inhibitor/Label	Structure	Inhibitor/Label	Structure
DU-UC15 (2c) **T97**	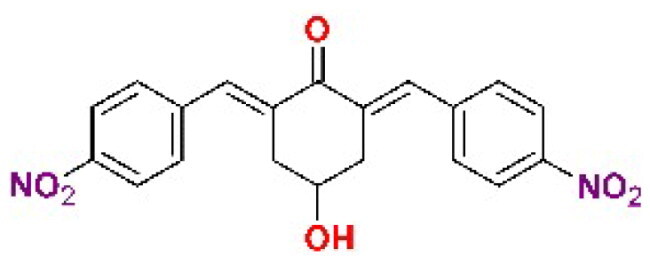	**T121**	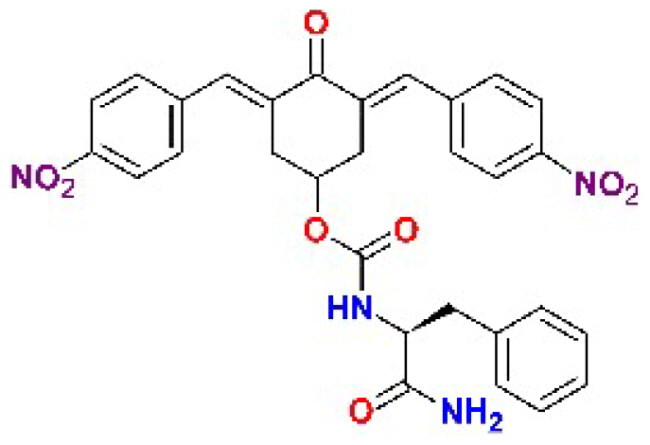
**T98**	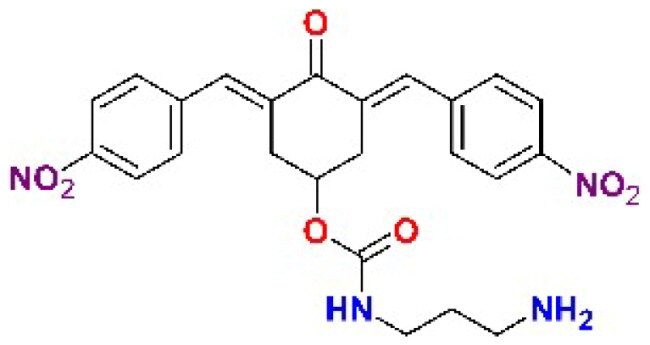	**T122**	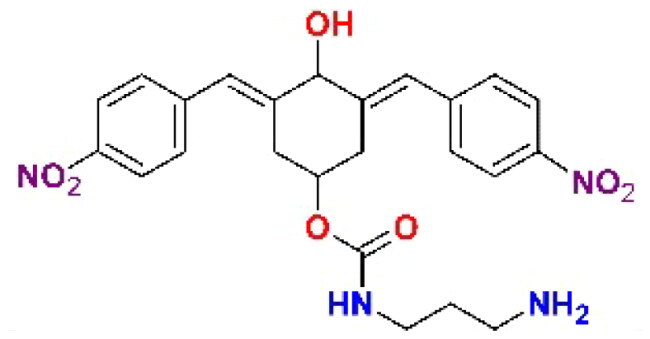
**T105**	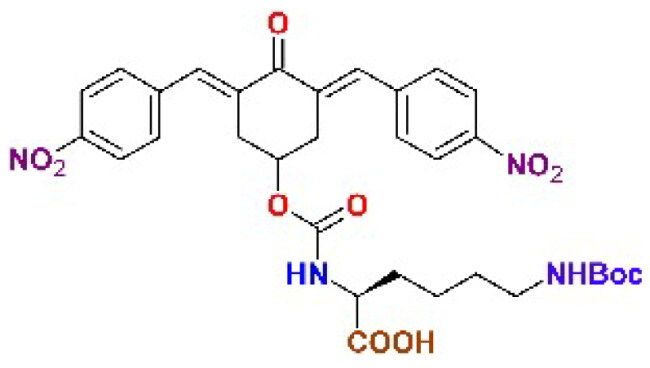	**T123**	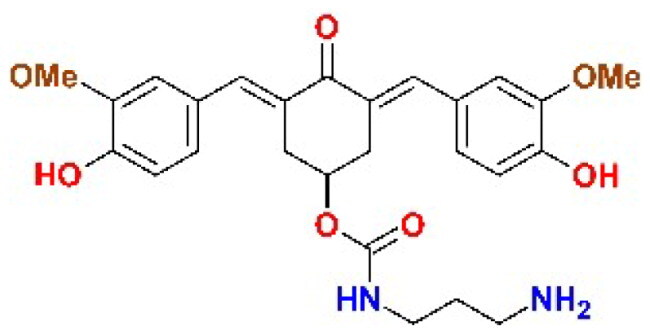
**T106**	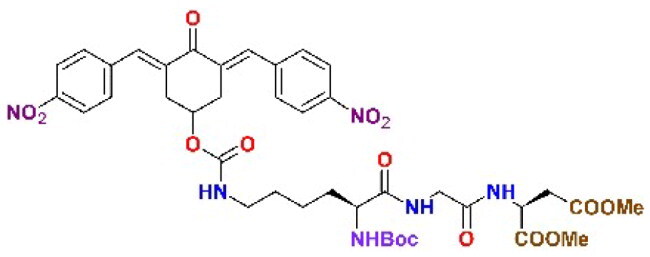	**T124**	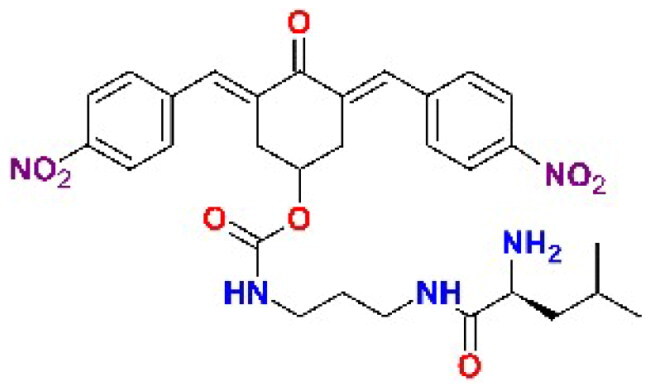
**T120**	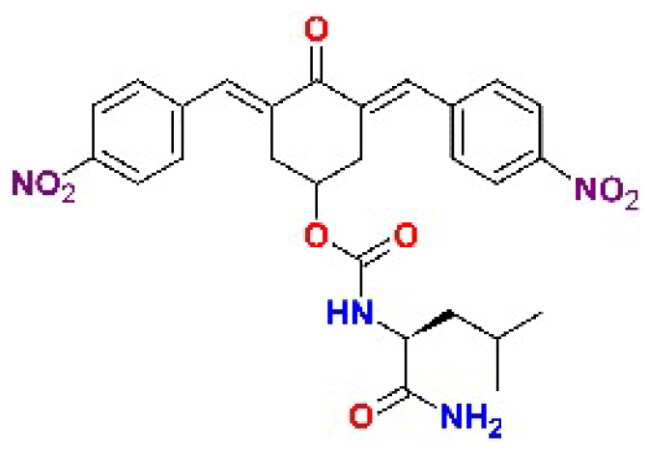	**T125**	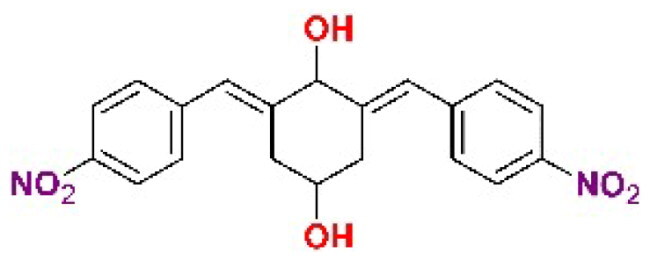

**Scheme 1.**
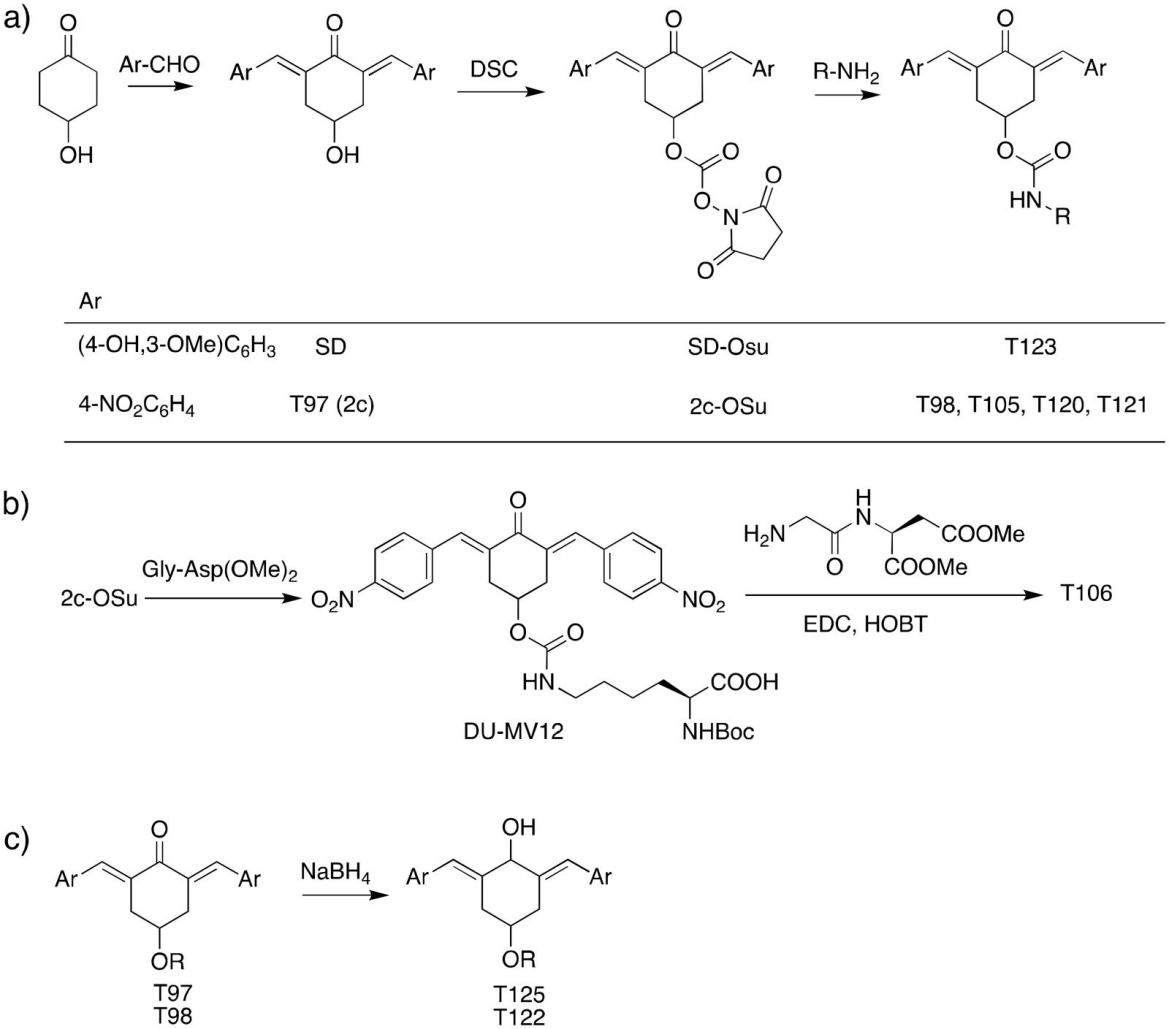
Synthesis of bis(benzylidene)cyclohexanone inhibitors.

The general approach to the synthesis of BBC starts with aldol condensation of *4*-hydroxycyclohexanone with the appropriate aromatic aldehyde, after which the *4*-hydroxycyclohexanone scaffold is modified as indicated (Scheme 1).

Double condensation of *4*-hydroxycyclohexanone with *4*-nitrobenzaldehyde gives compound **T97**. Compounds **T98, T105, T120 and T121** were synthesised from the carbonate *2c*-OSu ^32^ by reaction with the appropriate primary amine as shown in Scheme 1a. Similarly, the reaction of *2c*-OSu with *N*-Boc-Lys gave compound DU-MV12 from which **T106** was obtained by condensation with the dipeptide Gly-Asp(OMe)_2_ (Scheme 1b). Compound **T123** was obtained by aldol condensation of *4*-hydroxycyclohexanone with vanillin under acidic conditions, followed by carbonylation of the resulting alcohol with *N,N’*-disuccinimidyldicarbonate (DSC) and reaction with *1,3*-diaminopropane. **T125** and **T122** were obtained by borohydride reduction of **T97** and **T98**, respectively (Scheme 1c).

The synthesis of compounds **T97** (DU-UC15, 2c), **T98** and **T124** has previously been reported (**T97**^32^; **T98**, **T124**^34^). Full details of the syntheses of **T120** and **T121** will be reported elsewhere.

**T105**. A solution of 2c-OSu (0.639 g, 1.22 mmol) and ^ε^N-Boc-Lys (0.351 g, 1.47 mmol) in dichloromethane was adjusted to pH 8–9 with triethylamine and stirred for 12 h until complete conversion was observed (TLC). The reaction mixture was extracted with 20% aqueous citric acid and the organic layer was dried over Na_2_SO_4_ and evaporated to obtain the desired product. Yield 83%.^1^H NMR (CDCl_3_, 500 MHz) δ (ppm): 8.27–8.24 (*m*, 4H), 7.92–7.84 (*m*, 2H), 7.59–7.54 (*m*, 4H), 5.43 (*m*, 1H), 5.25–5.07 (*m*, 1H), 4.79–4.55 (*m*, 1H), 4.34–3.89 (*m*, 1H), 3.39–2.85 (*m*, 6H), 1.86–1.54 (*m*, 2H), 1.51–1.20 (*m*, 4H), 1.40 (*s*, 9H).^13^C NMR (CDCl_3_, 125 MHz) δ (ppm): 187.6, 175.6, 156.5, 155.2, 147.6, 141.7, 137.6, 134.4, 130.9, 123.9, 79.7, 67.8, 53.7, 40.0, 33.3, 31.6, 29.8, 28.5, 22.3. ESI-MS: m/z 675.2 [MNa]^+^, 691.2 [MK]^+^.

**T106**. 2c-OSu (0.856 g, 1.64 mmol) and ^α^N-Boc-Lys (0.458 g, 1.86 mmol), as described above for **T105**, gave the carbamate DU-MV12 (86%). ^1^H NMR (CDCl_3_, 500 MHz) δ (ppm): 8.30–8.26 (*m*, 4H), 7.93–7.87 (*m*, 2H), 7.62–7.57 (*m*, 4H), 5.24–5.12 (*m*, 1H), 5.07 (d, 1H, *J* = 7.3 Hz), 4.73 (t, 1H, *J* = 5.9 Hz), 4.32–4.04 (*m*, 1H), 3.31–2.93 (*m*, 6H), 1.86–1.57 (*m*, 2H), 1.53–1.24 (*m*, 4H), 1.42 (*s*, 9H). ^13^C NMR (CDCl_3_, 125 MHz) δ (ppm): 187.9, 175.9, 155.9, 155.5, 147.7, 141.7, 137.4, 134.6, 130.9, 124.0, 80.5, 67.2, 53.2, 40.6, 33.4, 32.0, 29.3, 28.5, 22.8. ESI-MS: m/z 675.2 [MNa]^+^, 691.2 [MK]^+^. This compound (0.484 g, 0.742 mmol) was stirred with EDC^.^HCl (0.213 g, 1.113 mmol) and HOBt (0.150 g, 1.113 mmol) in dichloromethane at 0 °C for 30 min. After 10 min, Gly-Asp dimethyl ester trifluoroacetate (0.256 g, 0.771 mmol) in dichloromethane was slowly added to the mixture together with the minimum volume of triethylamine to reach an apparent pH 8–9. The resulting solution was stirred at 25 °C for 18 h till complete conversion of DU-MV12 to the product (TLC) and the mixture was extracted with 20% aqueous citric acid (3 x) and sat. aqueous NaHCO_3_ (3 x). The organic layer was dried over Na_2_SO_4_ and evaporated *in vacuo* to obtain the crude product, which was purified by flash chromatography (eluent: CHCl_3_/MeOH from 100:0 to 97:3). Yield = 39% from DU-MV12; yellow solid. ^1^H NMR (CDCl_3_, 500 MHz) δ (ppm): 8.30–8.23 (*m*, 4H), 7.95–7.84 (*m*, 2H), 7.65–7.54 (*m*, 4H), 7.06 (d, *J* = 8.1 Hz, 1H), 6.61 (*t*, *J* = 5.1 Hz, 1H), 5.30–5.09 (*m*, 2H), 4.94 (*t*, *J* = 5.7 Hz), 4.82 (dt, *J* = 8.1, 4.7 Hz), 4.15–3.89 (*m*, 3H), 3.72 (*s*, 3H), 3.66 (*s*, 3H each), 3.33–2.91 (*m*, 7H) 2.83 (dd, *J* = 17.2, 4.7 Hz, 1H), 1.93–1.70 (*m*, 2H), 1.69–1.21 (*m*, 4H), 1.40 (*s*, 9H). ^13^C NMR (CDCl_3_, 125 MHz) δ (ppm) 187.7, 172.6, 171.4, 170.9, 168.6, 155.9, 155.4, 147.7, 141.7, 137.3, 134.6, 130.9, 124.0, 80.4, 67.1, 54.4, 53.1, 52.3, 48.7, 42.9, 40.5, 36.0, 33.4, 32.0, 29.3, 28.4, 22.6. ESI-MS: m/z 875.4 [MNa]^+^, 981.3 [MK]^+^.

**T123** (DU-SD2). Following the procedure described for **T105**, compound DU-SD2 was synthesised from 2c-OSu (0,752 g, 1.43 mmol) and 1,3-diaminopropane (0,128 g, 0,145 ml, 1,72 mmol). Yield 39%. ^1^H NMR (400 MHz, D_6_-DMSO) δ (ppm): 9.58 (*s*, 2H), 7.65 (*s*, 2H), 7.58 (*m*, 3H) 7.16 (br, 1H), 7.09 (*s*, 2H), 7.01 (apparent d, 2H), 6.85 (apparent d, 2H), 5.02 (br, 1H), 3.80 (*s*, 6H), 3.16 − 3.05 (*m*, 4H), 2.91 (*m*, 2H), 2.49 (*m*, 2H), 1.56 (*m*, 2H) ppm. ^13^C NMR (100 MHz D_6_-DMSO) δ (ppm): 187.55, 159.00, 148.48, 147.93, 138.62, 129.80, 126.99, 124.51, 116.05, 115.32, 56.14, 46.16, 37.65, 37.14, 33.22, 27.92.

**T125.** To a solution of **T97** (2c) (0.152 g, 0.4 mmol) in 25 ml 1:10 MeOH/THF, 0.017 g of NaBH_4_ (0.44 mmol) were added. The resulting solution was stirred at 25° C for 1.5 h until complete conversion to product (TLC). After adding 25 ml of brine, the mixture was extracted with diethyl ether (3 x). The organic layer was dried over Na_2_SO_4_ and evaporated *in vacuo* to obtain the product as a mixture of cis/trans diastereoisomers. Yield = 93%., ^1^H NMR (400 MHz, D_6_-DMSO) δ (ppm): 8.19, 8.16 (2d, 4H, *J* = 8.5 Hz), 7.57, 7.53 (2d, 4H, *J* = 8.5 Hz), 6.76, 6.74 (2*s*, 2H), 5.81, 5.73 (2d, 2H, *J* = 4.6 Hz), 5.04, 4.98 (2d, 2H, *J* = 4.6 Hz), 4.74, 4.66 (2 *m*, 2H), 3.86, 3.65 (2 *m*, 2H), 2.95, 2.75 (2dd, 2H, *J* = 4.0 Hz, *J* = 13.5 Hz), 2.56, 2.31 (2dd, 2H, *J* = 3.0 Hz, *J* = 13.5 Hz). ^13^C NMR (100 MHz, D_6_-DMSO) δ (ppm): 188.27, 148.22, 147.87, 137.73, 131.37, 127.36, 124.44, 115.97, 115.22, 64.11, 56.11, 36.58, 188.27, 148.22, 147.87, 137.73, 131.37, 127.36, 124.44, 115.97, 115.22, 64.11, 56.11, 36.58

**T122.** Reduction of **T98** (120 mg, 0.25 mmol), as described for **T125**, gave the product as a mixture of cis/trans diastereoisomers. Yield 67%. ^1^H NMR (500 MHz, D_6_-DMSO) δ (ppm): 8.18, 8.16, (2d, 4H, *J* = 8.7 Hz), 7.53, 7.51 (2d, 4H, *J* = 8.7 Hz), 7.11 (bt, 1H), 6.82, 6.79 (2*s*, 2H), 5.91, 5.86 (2 d, *J* = 4 Hz each, 1H), 5.83 (d, *J* = 4.0 Hz, 1H), 5.29 (2*t*, *J* = 2.1 Hz, 1H), 3.30 − 3.10 (*m*, 4H), 2.85 (*m*, 2H), 2.61 (bm, 2H), 1.53 (quint, 2H); ESI-MS: *m*/*z* 483.2 [MH]^+^, 505.1 [MNa]^+^.

### Determination of IC_50_ by fluorogenic enzyme assay kits

Half-maximal inhibitory concentrations IC_50_ of the compounds tested for SARS-CoV-2 M^pro^ and PL^pro^ were measured using commercially available kits from BPS Bioscience Inc. (San Diego, CA, USA), specifically SARS-CoV-2 M^pro^ untagged assay kit (catalog #78042–1), SARS-CoV-2 PL^pro^ assay kit, protease activity (catalog #79995–1) and SARS-CoV-2 PLpro assay kit, deubiquitinase activity (catalog # 79996), all in 96-well format. The assay kits come with purified recombinant enzymes (M^pro^ and PL^pro^), assay buffers and control inhibitors (GC376 for M^pro^ and GRL0617 for PL^pro^), and fluorogenic substrates. According to information from the manufacturer’s website (bpsbioscience.com), all substrates were the following quenched compounds. For SARS-CoV-2 M^pro^ assay it was 14-mer fluorogenic peptide (DABCYL-KTSAVLQSGFRKME-EDANS), and upon proteolysis by M^pro^ the peptide substrate is cleaved between glutamine and serine generating the highly fluorescent peptide fragment (SGFRKME-EDANS). For SARS-CoV-2 PL^pro^ protease activity assay it was 5-mer fluorogenic peptide (Z-RLRGG-AMC), and upon proteolysis by PL^pro^ fluorescent AMC dye (7-amino-4-methylcoumarin) is generated. For SARS-CoV-2 PL^pro^ deubiquitinase activity assay it was ubiquitin conjugated by C-terminal Gly with AMC (Ub-AMC), and upon proteolysis by PL^pro^ fluorescent AMC dye is generated. The manufacturer has recommended excitation/emission wavelengths for the determination of enzyme activity for all kits at 360/460 nm. The assay procedures were performed according to the manufacturer’s recommendations, shortly as follows. The inhibitors were screened against recombinant SARS-CoV-2 M^pro^ and PL^pro^ enzymes in the assay buffers delivered with the kits containing 1 mM DTT. The final concentration of DMSO in the assays did not exceed 1%. The steady-state measurements were started by adding the substrate solution to the wells and the incubation with slow shaking lasted for 4 h at room temperature (M^pro^ assay), or for 1 h at 37 °C (PL^pro^ assays). The fluorescence intensity was measured by a microtiter plate-reading fluorimeter BioTek Synergy HT (BioTek, Winooski, VT, USA). Enzyme activity was defined as the amount of enzyme that converts 1 µmol of substrate per minute. Data were fitted and IC_50_ values and the corresponding K_i_ values were calculated from the Dixon plot using GraphPad Prism 9.5.0 (GraphPad Software, LLC).

In addition to the BBC studied, we have included a set of known cysteine protease inhibitors and proteasome-associated deubiquitinating enzyme inhibitors as reference compounds: RA-9^30^, PR-619 (**C4**)^46^^,^^47^, RA-190^30^, GRL0617 (**C9**)^48^, GC376 (**C10**)^49^^,^^50^ (Table 2). The table also includes an additional reference inhibitor remdesivir (**C8**)^51–54^ with different mechanism of action, thus was added later for the cellular assay as a control for SARS-CoV-2 inhibition.

**Table 2.** Chemical structures of the reference inhibitors of M^pro^ and PL^pro^ of SARS-CoV-2 or proteasome-associated deubiquitinating enzyme inhibitors included in this study.

Inhibitor/Label	Structure	Inhibitor/Label	Structure
PR-619 **C4**	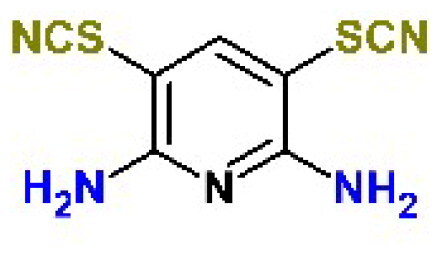	Remdesivir **C8**	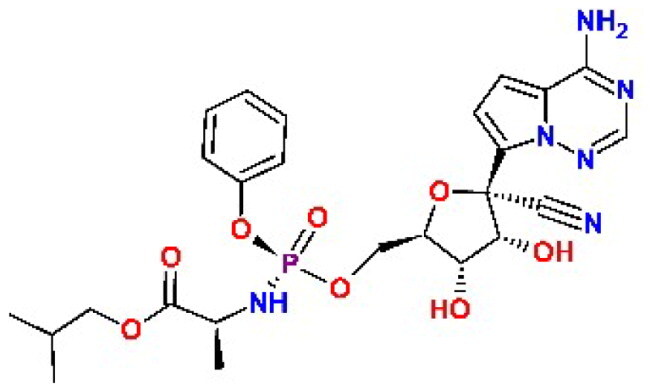
GRL0617 **C9**	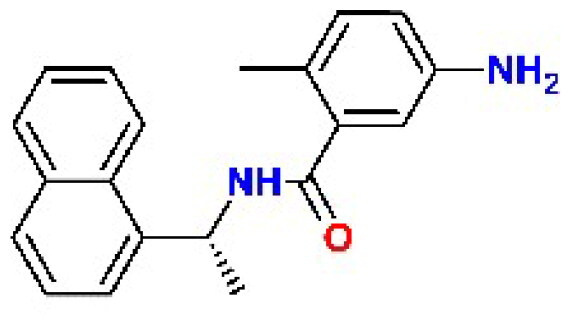	GC376 **C10**	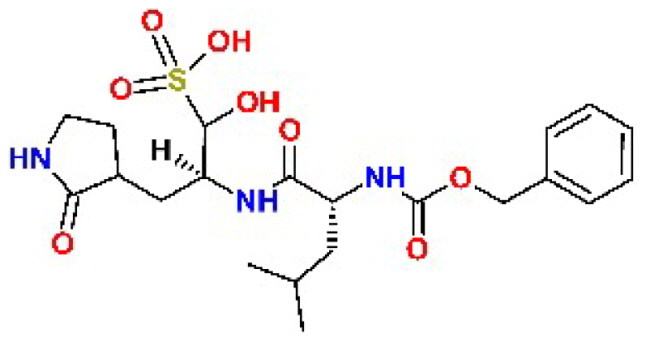
RA-9	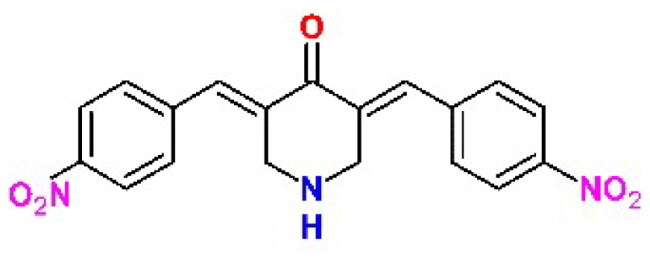	RA-190	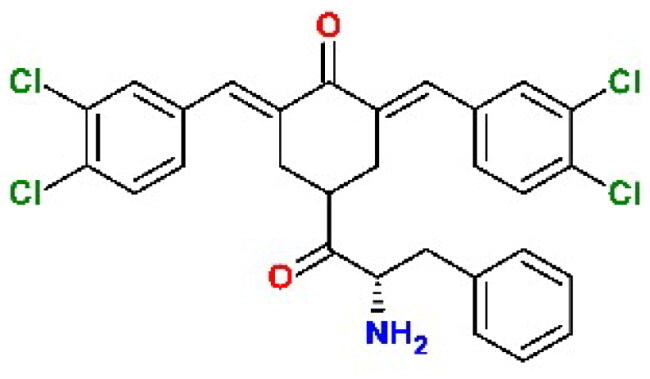

### Compounds stocks

Stock solutions of individual compounds in solid form (10 mg/ml, at approx. 10–50 mM concentrations depending on the individual M_w_) were prepared by dissolving in sterile dimethyl sulfoxide (DMSO) and filtered through a 0.2 µm filter (4 mm syringe filter) for fluorogenic enzyme assays. For cell lines, the stock solutions containing DMSO were diluted to the 1 mM concentration in PBS buffer. All stock solutions were kept under dark conditions and kept at −20 °C. For the cytotoxicity and plague reduction assay, stocks were further diluted to appropriate concentrations ranging from 0.1 µM up to 30 µM in cell condition medium.

### Virus

The original SARS-CoV-2 strain was isolated from a clinical sample of a patient with COVID-19 in Slovakia. The isolate was deposited in the European virus archive (EVA) GLOBAL. The Slovak strain called Slovakia/SK-BMC5/2020 (available at https://www.european-virus-archive.com/virus/sars-cov-2-strain-slovakiask-bmc52020) represents the strain circulating in Europe in spring 2020 and carries the Spike D614G mutation (lineage B0.1). The identity of the virus was confirmed by sequencing. The complete genome sequence was deposited in the GISAID.org database under the accession ID EPI_ISL_417879. The virus was isolated from a clinical sample, passaged and propagated in VERO-E6 cells, and the presence of mycoplasma was tested with a negative result before its use in this study.

### Establishment of a workflow for in vitro biological evaluation

To establish the effective workflow for the evaluation of therapeutic potential, we sought to optimise the testing strategy in several steps. As the first step in biological evaluation, we selected model cell lines based on their known enzymatic characteristics and the level of available data on the RNA expression of relevant genes (e.g., angiotensin converting enzyme-2 (ACE2) entry receptor, transmembrane protease serine 2 (TMPRSS2), and others). Several different types of host cells have been used in SARS-CoV-2 infection assays, as well as for determination of viability and cytotoxicity; therefore, the following models were considered in our studies: VERO-E6^55^, Caco-2^56^, PK-15^57^, and A549^58^. The most common cell type recently used in coronavirus-related studies are primate-derived epithelial kidney VERO-E6 cells due to their ability to propagate SARS-CoV-2 very effectively^55^. Short-time SARS-CoV-2 infection was reported to produce relevant cytopathic effects in the VERO-E6 cell line. However, since VERO-E6 cells are not an accurate mimic of human airway and lung epithelial cells, the primary site of SARS-CoV-2 infection, we also conducted the experiment with other parallel cell lines of human origin. Two of these model systems are Caco-2 and A549, of which the first has the more sufficient expression levels of ACE2 to allow efficient infection with SARS-CoV-2. Caco-2 is an immortalised human colorectal adenocarcinoma cell line that is a primary model of the human intestinal epithelial barrier and has higher expression of the RNA serine protease TMPRSS2, which is known to mediate pathways of coronavirus infection; for example, priming of SARS-CoV-2 S protein^7^. A549 cells are more relevant to the primary infection site of coronavirus since they represent a human pulmonary epithelial cell model used to study drug metabolism^58^. However, these cells do not maintain sufficient levels of ACE2. The last cell line used in our studies is the mammalian-origin PK-15 model, which is the kidney epithelial cell line and serves as a good model for the multiplication of various mammalian viruses while studying host immune responses^57^. Due to the individual specificities of the cell models, we sought to evaluate the cytotoxicity of the compounds in all of them, while for the evaluation of antiviral efficacy we report the data collected in VERO-E6 cells only, while including some gathered remarks from other models.

The second important step was to estimate the exposure interval to cover the full effect of the compounds studied. As the compounds tested are expected to act as inhibitors of SARS-CoV-2 viral proteases, we set the exposure interval of the compounds at least 72 h after 3 h of incubation with SARS-CoV-2 alone. The last parameter to set was the multiplicity of infection (MOI). The cells were infected at low MOI to achieve more replication cycles. Together, the fully optimised strategy for this biological evaluation shown here (Figure S1) allowed us to thoroughly test and analyse the inhibitory potency of new BBC.

### Virus titre determination

Classical plaque assays, according to De Madrid *et al.*^59^, were performed in VERO-E6 cells grown at 37 °C and 5% CO_2_ in DMEM supplemented with 5% FBS and 2 mM L-glutamine. In summary, cells were seeded 24 h prior to virus titration to receive around 80% confluency. The next day, serial 10-fold dilutions of virus were added to the cells. After 1 h, the suspension was washed with PBS and overlaid with 1.5% (w/v) CMC in DMEM. Following a 4-day incubation at 37 °C and 5% CO_2_, cells were fixed for 20 – 30 min with formalin and plaques were visualised by staining with 0.5% crystal violet (1.01408.0025, Merck) at room temperature for 10 – 20 min. After washing the wells with water, the number of plaques was counted. The virus titre was expressed as plaque-forming units (PFU) per ml. The virus stocks were kept in the dark at −80 °C.

### Measurement of cytotoxicity and cell viability

For the cell cytotoxicity assay, A549, VERO-E6, Caco-2, and PK-15 cells were seeded into 96-well plates (10 000 cells of VERO-E6, PK-15, and A549 and 40 000 cells of Caco-2). Cells were cultured overnight at 37 °C in a humidified atmosphere. Cells were grown 72 h after the addition of individual compounds in appropriate concentrations (0.5, 1.0, 5.0, 10, 15, and 30 µM) and followed by microscopy inspection. Untreated cells were considered negative control, and DMSO treated cells were considered vehicle control. The classical MTT assay^60^ was used to measure cellular metabolic activity as an indicator of cell viability, proliferation, and cytotoxicity. Briefly, this colorimetric assay is based on the reduction of a yellow tetrazolium salt (dimethylthiazol-diphenyltetrazolium bromide, MTT) to purple formazan crystals by metabolically active cells. The viable cells contain oxidoreductase enzymes that reduce MTT to formazan thus resulting in a coloured solution that is quantified by measuring absorbance at 570 nm using a spectrophotometer. The darker the solution, the greater the number of viable, metabolically active cells. The results were plotted in a graph of cell viability (represented by a fold change normalised to an untreated control) against compound concentrations. All experimental and reference compounds were measured using at least 2 replicates for each.

### Quantitative SARS-CoV-2 plaque reduction assay

The plaque reduction assay with the SARS-CoV-2 virus (performed in a Biosafety level 3 containment laboratory BSL3 at the Biomedical Research Centre of the Slovak Academy of Sciences) was used to determine the inhibition capacity of several new and commercially available reference compounds. The SARS-CoV-2 virus at multiplicity of infection (MOI) = 1.0 was added to the monolayer of VERO-E6 cells (at a concentration of 2.5 x 10^5^ cells per well seeded overnight) in 12-well plates and incubated at 37 °C for 3 h. Serial dilutions (0.1, 0.25, 0.5, 1, 2.5, 5, 10, 15, and 30 µM) of compounds in DMSO-PBS-DMEM solution were then added to SARS-CoV-2 infected VERO-E6 cells and incubated at 37 °C for another 72 h. Cells with supernatant were collected, stored at −80 °C and virus titre was determined by plaque assay as described above. The results were plotted in a plaque reduction represented by the normalised response of PFU/ml to the untreated control against compound concentrations. The inhibition activity (represented by EC_50_) was determined from the normalised response plotted against logarithmic compound concentrations as the reciprocal of the highest dilution resulting in a 50% infection reduction. Data were processed by regression analysis and fitted to a logistic 4-parameter sigmoidal dose response curve using GraphPad Prism 9.5.0 (GraphPad Software, LLC). The goodness of fit is represented by the R^2^ parameter (for most experimental compounds, the data fit the model very well with R^2^ ∼ 0.85–0.91).

### Molecular modelling and in silico screening

**Figure 2.**
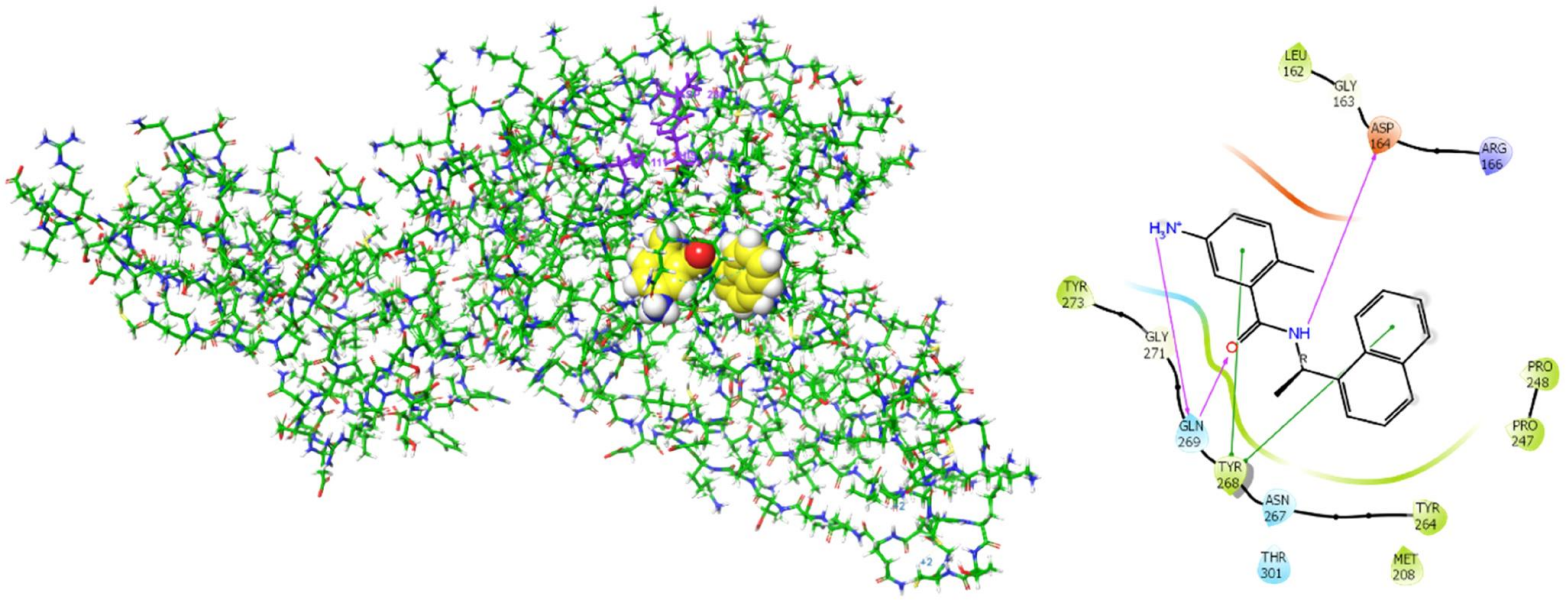
**Left:** 3D structure of the SARS-CoV-2 PL^pro^-GRL0617 complex (PDB entry 7CMD^62^). The inhibitor GRL0617 that occupies the S_4_ - S_3_ pockets of the substrate binding cleft is shown in the CPK representation. Catalytic triad of PL^pro^: Cys111 - His272 - Asp286 coloured purple shows the location of the catalytic site. **Right:** 2D scheme of inhibitor - residue interactions. Binding of GRL0617 induces closure of the flexible blocking loop BL2 (Gly266 - Gly271) and narrows the substrate binding cleft. The residues Tyr268 and Gln269 in the BL2 loop shift towards the bound GRL0617.

BBC with observed deubiquitinating activity towards the analogous SARS-CoV^22–25^ were also studied by molecular modelling. We have modelled inhibitor-enzyme interactions at the ligand binding site using the crystal structure of the PL^pro^-GRL0617 complex available from the Protein Data Bank^61^ (PDB entry 7CMD^62^) shown in Figure 2. GRL0617 is a potent noncovalent inhibitor of SARS-CoV-2 PL^pro^ (IC_50_ = 2.1 µM)^48^ that shows the highest structural similarity to the studied BBC among known PL^pro^ inhibitors with the published crystal structure of the enzyme-inhibitor complex. Previously, potent PL^pro^ inhibitors were derived from GRL0617, which occupies the BL2 groove of the PL^pro^ binding site (i.e., S_4_ - S_3_ pockets) and prevents substrate binding. These covalent inhibitors enhance the methyl group of the benzamide ring using a Gly-Gly-mimetic linker that fills the narrow S_2_ - S_1_ substrate channel and an electrophilic warhead that extends to the catalytic Cys111^63^. We have modelled the formation of a noncovalent enzyme-inhibitor complex (E:I **1**, Figure 1) that precedes the eventual formation of a covalent adduct to the catalytic cysteine^64^. BBC were docked to the GRL0617 binding site using extra precision algorithm of Glide^65^ (Schrödinger, LLC, New York, NY, 2021, release 2021–2). The ligand and water molecules were removed from the binding site, which was defined as the space that covers the S_4_ and S_3_ binding pockets of the PL^pro^ located near the catalytic centre. PL^pro^ cleaves the non-structural viral proteins nsp1-nsp4, as well as human ubiquitin and ISG15^1^ with strong specificity for the sequences L(R/K)GG↓(A/K), respectively, indicating that the S_2_ and S_1_ pockets of the active site are narrow and consequently cannot accommodate bulkier inhibitors alone^13^. The zinc ion was retained in the crystal structure of PL^pro^. We have validated the docking protocol by performing redocking of GRL0617, which resulted in a pose of the ligand very similar to that of the crystal structure. The PL^pro^-inhibitor complexes generated were subsequently energy optimised by molecular mechanics using the OPLS4 force field^66^ considering the effect of solvent^67^ in Maestro (Schrödinger, LLC, New York, NY, 2021, rel. 2021–2). The predicted enzyme-inhibitor (E:I) binding energies of the associate: $\Delta E_{\text{int}}=E_{\text{tot},\text{MM}}(E:I)‐\;E_{\text{tot},\text{MM}}(E)‐\;E_{\text{tot},\text{MM}}(I)$ calculated with help of molecular mechanics (MM) total energies ($E_{\text{tot},\text{MM}}$), were compared with the determined IC_50_ values of the enzyme assays on PL^pro^ inhibition. Several laboratories adopted a similar approach to the *in silico* identification of SARS-CoV-2 PL^pro^ inhibitors among natural compounds and drug repurposing^27^^,^^28^. To assess the ability of the BBC studied to undergo a nucleophilic attack of the thiol group of catalytic cysteine, we have calculated the Mulliken net atomic charges^68^ of the carbon C* of the benzylidene double bond as well as LUMO^2^ energies of the optimised geometries of studied compounds (Figure 1) using density functional theory (DTF-M06-2X/6–31++G** method)^69^ in Jaguar (Schrödinger, LLC, New York, NY, 2021, release 2021–2).

### Statistical analysis

Statistical methods used for the evaluation of the data are described in the figure legends. The statistical significance of means ± STDEV was evaluated using the two-tailed Student’s *t*-test. For all statistical analyses, *p* values < 0.05 were considered significant. All evaluations were performed using GraphPad Prism 9.5.0 (GraphPad Software, LLC), if other software was not explicitly mentioned.

## Results and discussion

### Validation of methods for determination of inhibitory potencies and effectivities

The half-maximal inhibitory concentrations IC_50_ of the selected reference inhibitors (Table 2) towards M^pro^ and PL^pro^ of SARS-CoV-2 were determined by enzyme assays. Table 3 shows the half-maximal IC_50_ and EC_50_ determined for selected reference inhibitors. The half-maximal effective *in vitro* antiviral concentrations (EC_50_) measured here by the *in vitro* plaque reduction assay in VERO-E6 cells (e.g., GC376 (**C10**) showed EC_50_ of 1.0 µM) correspond well to the values reported in the literature (EC_50_ of 3.37 µM)^50^ obtained by performing a similar assay. Likewise, inhibitory concentrations IC_50_ measured here by fluorogenic enzyme assays (e.g., for GRL0617, the IC_50_ of 3.2 µM) are comparable to IC_50_ obtained from the literature (e.g., GRL0617 IC_50_ of 1.37 − 2.10 µM) for inhibition of protease activity^48^^,^^62^. Furthermore, the measured inhibitory concentrations IC_50_ for reference inhibitors that showed inhibitory potency in enzymatic assays (PR-619, GRL0617, and GC376) are lower, but comparable to their measured antiviral effective concentrations EC_50_ (Table 3). In the case of the inhibitor PR-619 (**C4**), we observed the inhibitory effect on both M^pro^ and PL^pro^ (both protease and deubiquitinase activities), and here we find that inhibitory concentrations IC_50_ are lower than those obtained from the literature^46^. However, such differences are common when comparing results of different enzymatic assays using different substrates and different sources of enzymes. Bis-benzylidene piperidone RA-190 binds directly and covalently to Cys88 of the RPN13 Pru ubiquitin receptor domain in the regulatory particle 19S and inhibits proteasome function, triggering rapid accumulation of polyubiquitinated proteins^70^. RA-9 is a non-specific inhibitor that irreversibly inhibits DUBs by exposing its carbonyl group to a nucleophilic attack from the cysteine -SH group^71^. Furthermore, the inclusion of remdesivir (**C8**) in our study was not a random choice. Today, most *in vitro* efficacy studies have reported positive results on remdesivir (**C8**) anticoronaviral activity^51–54^. Therefore, this commercially available compound was included as a positive control for the validation of the method in our studies on infected human cells. Remdesivir did not show any inhibitory effect on M^pro^ and PL^pro^ of SARS-CoV-2 (Table 3) since its mechanism of antiviral activity is related to the viral RNA-dependent RNA polymerase. Strangely enough, some authors in the recent literature^72^ came to a wrong conclusion based on molecular simulations claiming that remdesivir is an efficient inhibitor of M^pro^. In any case, the effective antiviral concentration of remdesivir measured here in cells infected with SARS-CoV-2 (EC_50_ of 1.8 µM) corresponds well to the data (EC_50_ of 0.77 µM) reported in^54^ (Table 3). Thus, all these findings collectively validate the methods used in this work to determine the antiviral activities of the BBC.

**Table 3.** Determined IC_50_ and EC_50_ and cytotoxicity of selected reference inhibitors of cysteine proteases.

Label	Protease inhibitor	IC_50_ (µM)				**CTE** * **(µM)**	EC_50_ (µM)		(Ref.)
		**Reference value** **M^pro^/ PL^pro^ target**	Enzyme assay			Cell viability MTT assay			
			**PA** *		**DUA** *				
			M^pro^	PL^pro^	PL^pro^		Reference value	Plaque reduction assay	
**C4**	PR-619	6.1 − 12.9*	0.7	0.1	0.2	>10.0	n/a	9.0	(a, b)
**C8**	Remdesivir	n/a	> 100	> 100	> 100	n/o	0.77 − 0.99^#^	1.8	(c, d)
**C9**	GRL0617	1.37 − 2.10*	> 100	3.2	2.0	n/o	n/a	2.5^‡^	(e, a)
**C10**	GC376	0.03 − 0.15	0.02	> 100	>100	n/o	3.37^#^	1.0	(f, g)

* **Abbreviations used**: Deubiquitinase activity (**DUA**); protease activity (**PA**); compound concentration that cause >40% reduction in the number of viable cells (**CTE**) averaged on screened cell lines; no cytotoxic effect observed within the concentration range (**n/o**); reference value is not available (**n/a**) in literature or the data range not measured; literature reference (**Ref**); ^#^ measured *in vitro* on virus infected cells or cell-free assay; ^‡^ value out of acceptable range of R^2^ correlation coefficient.

**References used**: (**a**) ^46^; (**b**) ^73^; (**c**) ^54^; (**d**) ^74^; (**e**) ^48^^,^^62^; (**f**) ^50^; (**g**) ^75^.

### Determination of cytotoxic effect of studied compounds

To determine the actual cytotoxicity *in vitro* of the biologically active compounds listed in Table 1, we tested cellular viability using MTT assay in multiple cell lines. Additionally, a series of commercially available reference inhibitors listed in Table 2 were tested in parallel to validate the method. The four model cell lines VERO-E6, Caco-2, PK-15, and A549 were used as described above. Cells were incubated with each compound at concentrations ranging from 0.5 to 30 µM and any possible cytotoxic effect was monitored for 72 h. Novel compounds were dissolved in dimethyl sulfoxide (DMSO), and compounds showing any signs of insolubility were excluded from the analysis. The cytotoxic effect (CTE) is represented here as the concentration that causes a reduction of more than 40% in number of viable cells (Figure S2). Cell viability results were averaged for all screened cell lines and are shown in Tables 3-4 and Figure S2. Interestingly, all experimental compounds were relatively well tolerated in selected cell lines, except **T97**. This was considered cytotoxic even at lower concentrations (starting at approximately 5 µM) in the VERO-E6 cell line. Our findings for **T97** (DU-UC15) are truly in line with its previously reported antiproliferative properties in several models, while an analogue of **T97** modified for elevated solubility and bioavailability shows anticancer activity *in vivo*^32–35^. However, considering also the small CTE of DMSO itself (shown at the end of graphs B-E, Figure S2), compound **T97** was excluded from further biological evaluations with live virus. The most pronounced cytotoxicity was observed in the case of the commercial inhibitors PR-619 and RA-190 that exhibited higher CTE at concentrations of approximately 5–10 µM over all cell lines.

**Table 4.** Determined IC_50_ and EC_50_ values of bis(benzylidene)cyclohexanones **T97** - **T125** and reference inhibitors of proteasome-associated deubiquitinating enzymes **RA-9** and **RA-190**.

Label	Molecular weight (Mw)	IC_50_ (µM)			CTE (µM)	EC_50_ (µM)
		Enzyme assay				
		PA		DUA		
		M^pro^	PL^pro^	PL^pro^ target	Cell viability MTT assay	Plaque reduction assay
**T97**	380.4	>100	>100	>100	>5.0	n/a
**T98**	481.5	>100	50	75	>15.0-30.0	6.7
**T105**	652.7	>100	>100	>100	n/o	n/o
**T106**	852.8	35	40	60	>30.0	n/o
**T120**	536.5	>100	90	85	>10.0	3.4
**T121**	570.2	>100	>100	>100	>10.0-15.0	8.4
**T122**	482.5	>100	60	75	>30.0	17.0
**T123**	382.4	>100	60	75	>30.0	n/o
**T124**	593.6	>100	>100	>100	>30.0	15.9
**T125**	382.4	>100	85	45	n/o	n/o
**RA-9**	365.3	>100	>100	>100	>30.0	12.1^‡^
**RA-190**	596.8	>100	>100	>100	>5.0	3.0

**Abbreviations used**: Deubiquitinase activity (**DUA**); protease activity (**PA**); compound concentration that causes more than 40% reduction in number of viable cells (**CTE**) averaged over screened cell lines; no cytotoxic or antiviral effect observed within the concentration range (**n/o**); value not available (**n/a**) due to compound’s higher cytotoxicity, insolubility, or data range not measured; ^‡^value out of acceptable range of R^2^ correlation coefficient.

These findings correspond very well to previously reported *in vitro* experiments that showed that PR-619 could decrease cell viability, mainly by inducing caspase3/7-dependent cell apoptosis^73^. Furthermore, Anchoori reported that compound RA-190 is selectively toxic to cells^70^. Although PR-619 and RA-190 appear to be unsuitable as biological probes due to their widespread reactivity^30^, such compounds could be profoundly cytotoxic to apoptosis-resistant tumour cells. On the other hand, compounds **T98**, **T120,** and **T121** were better tolerated in the human adenocarcinoma cell line Caco-2 and A549 (Figure S2-E) compared to epithelial kidney VERO-6 and PK-15 cells. In this case, **T98** exhibited very mild CTE at concentrations >15 µM and similarly **T120** and **T121** showed CTE at concentration >10–15 µM.

In conclusion, all compounds that exhibited cytotoxicity only at higher concentrations (CTE >15 µM) or had no observed cytotoxic effect within the screened concentration range (e.g., **T105** and **T125**), were further subjected to antiviral efficacy testing and estimation of EC_50_ in the presence of live SARS-CoV-2 virus. On the other hand, selectivity index (SI = CTE/EC_50_) of the most active compounds is low.

### Antiviral efficacy of new compounds against live SARS-CoV-2 isolate

To investigate the therapeutic potential of our newly synthesised inhibitors **T97**, **T98**, **T105**, **T106**, **T120-T125,** all compounds that passed the cytotoxicity requirements (CTE >15 µM in the VERO-E6 cell line) were tested for inhibition of SARS-CoV-2. EC_50_ inhibitory concentrations were measured by the *in vitro* plaque reduction assay in VERO-E6 cells. Known cysteine protease and deubiquitinase inhibitors were included in this study for comparison or as a reference: RA-9^30^; PR-619 (**C4**)^46^^,^^47^; RA-190^30^; remdesivir (**C8**)^51–54^; GRL0617 (**C9**)^48^; GC376 (**C10**)^49^^,^^50^.

Typical outcomes measured in such *in vitro* studies include the amount of virus (e.g. copies of certain ORF, or viral RNA); the number of virus-infected cells in the culture (e.g., number of plaque-forming units, PFU) or changes in viral replication rates. Therefore, in our biological experiment, the reduction in viral titre (in PFU/ml) was monitored and used to evaluate the inhibitory effect (EC_50_) against the live SAR-CoV-2 virus. For the BBC studied, the following inhibitory concentrations were determined: **T98** EC_50_ = 6.7 µM, **T120** EC_50_ = 3.4 µM, **T121** EC_50_ = 8.4 µM, **T122** EC_50_ = 17.0 µM, and **T124** EC_50_ = 15.9 µM, respectively (Figure 4, Table 4). Three new compounds **T98**, **T120** and **T121** show an inhibitory effect *in vitro* similar or higher than the reference compounds with similar chemical structures RA-9 and RA-190, as well as GRL0617 (**C9**) (Table 3, Figure S3). The compound **T120** showed the lowest EC_50_ value among the experimental compounds tested and represents bis(benzylidene)cyclohexanone with the highest inhibitory effect on the SARS-CoV-2 virus *in vitro*, while its CTE remains mild. In comparison, **T98** exhibited inhibition of SARS-CoV-2 in the same plaque reduction assay with the EC_50_ of 6.7 µM, which is approximately 2-fold weaker than **T120;** however, **T98** was better tolerated by model epithelial cells (e.g., in VERO-E6 was CTE >15 µM vs. >10 µM, respectively). Therefore, **T98** is also considered in this study a promising compound with the highest inhibitory effect against the SARS-CoV-2 virus *in vitro.* On the other hand, for the experimental compounds **T105**, **T106**, **T123** and **T125,** no antiviral effect was observed within the concentration range (0.1–30.0 µM) tested. Furthermore, the antiviral effect of known reference cysteine protease inhibitors determined in this work corresponds well to the EC_50_ available in the literature (Table 3)^49^^,^^50^^,^^54^^,^^74^. Although antiviral effect was observed for the six reference inhibitors (Table 3), EC_50_ was successfully determined for four of them: **C4**, **C8**, **C9,** and **C10**. The last two inhibitors RA-9 and GRL0617 (**C9**) give EC_50_ values outside the acceptable R^2^ range (Figure S3). This was perhaps due to a minimal number of points analysed and/or the concentration scale being out of range.

When comparing the observed EC_50_ of our three most promising experimental compounds, **T98**, **T120**, **T121,** with the most potent reference inhibitors included in this study, GC376 (**C10**) and remdesivir (**C8**) (Table 3), the experimental compounds showed *in vitro* efficiency EC_50_ against SARS-CoV-2 somewhat inferior, that is, approximately 3-4 times weaker (EC_50_ of 3.4 − 8.4 µM vs. 1.0 − 1.8 µM, respectively). However, both GC376 (**C10**)^49^^,^^50^ and remdesivir (**C8**)^51–54^ represent a different mechanism of inhibition of the virus than the inhibition of PL^pro^ proposed for the novel compounds. Although remdesivir was used as a positive reference for virus depletion, the GC376 inhibitor was chosen as a reference for viral protease activity.

Finally, the experimental plaque reduction assay correlated with evidence collected from the MTT viability assay, identified 3 (out of 10) new compounds with potent antiviral activity against SARS-CoV-2 in the concentration range as low as EC_50_ = 3.4 µM.

### Determination of inhibitory potencies of bis(benzylidene)cyclohexanones in enzymatic assays

**Figure 3.**
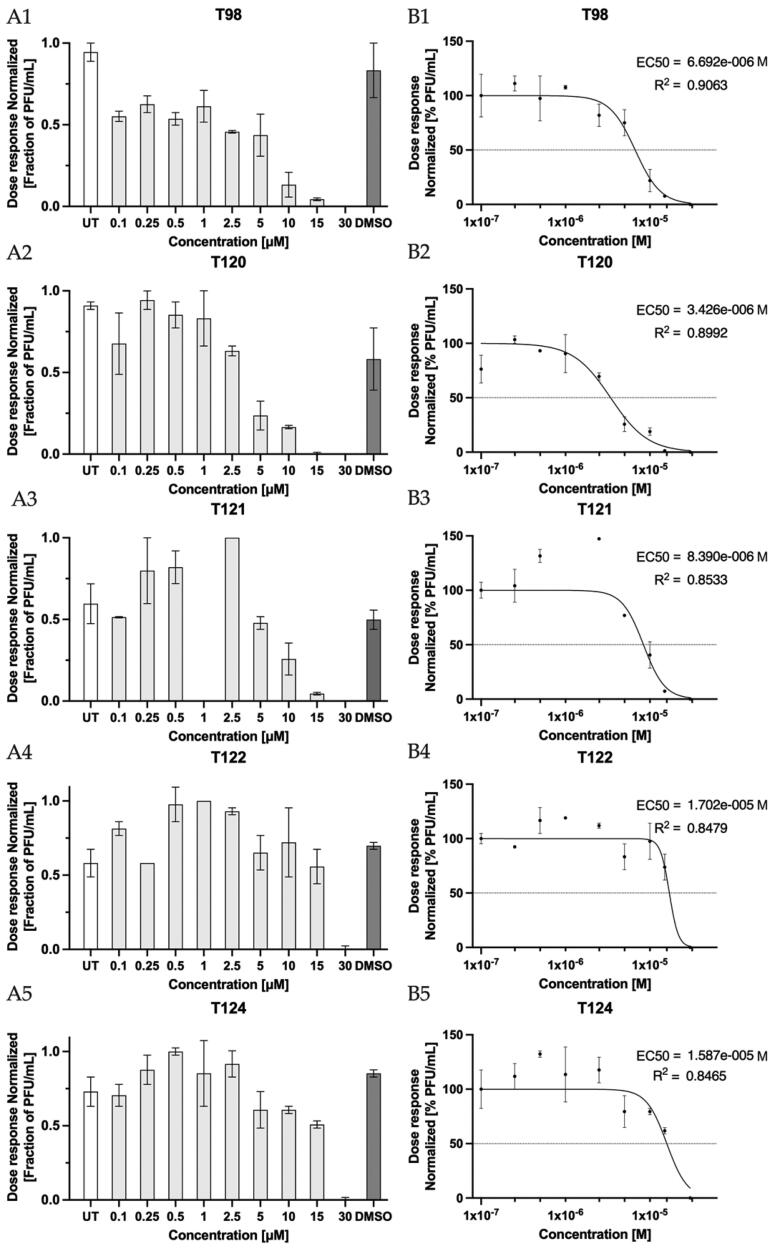
Evaluation of antiviral efficacy with calculation of the effective inhibitory concentration EC_50_ for compounds: **T98** (**A1, B1**), **T120** (**A2, B2**), **T121** (**A3, B3**), **T122** (**A4, B4)** and **T124** (**A5, B5**). Monitoring the trend of decreasing the SARS-CoV-2 virus titre (normalised to untreated control, presented as mean with SEM) with increasing compound concentration (**A1-A5**). Determination of half-maximal effective concentrations EC_50_ [M] (**B1-B5**) from the sigmoidal model with the corresponding regression coefficient R^2^ (the goodness of fit). Data (**B1-B5**) are presented as mean and error with SEM.

We did not find any reported information on inhibitory concentrations IC_50_ for reference proteasome-associated deubiquitinating enzyme inhibitors RA-9 and RA-190 measured using enzyme assays towards M^pro^ or PL^pro^ of SARS-CoV-2, and we did not observe any inhibitory effect for these two inhibitors (IC_50_ >100 µM, Table 4). The IC_50_ values of BBC were determined using the same enzymatic assays against M^pro^ and PL^pro^ of SARS-CoV-2 as were used for the reference inhibitors (Table 3). Of the 12 compounds tested, we observed some inhibitory effect (IC_50_ < 100 µM) in 50% of them (**T98**, **T106**, **T120**, **T122**, **T123**, and **T125**), Table 4 and Figure 3. In all cases, the compounds inhibited the protease and deubiquitinase activities of PL^pro^. Only compound **T106** also showed inhibitory potency towards the protease activity of M^pro^. All the IC_50_ values for the compounds tested fall in the range of 35-90 µM, so they were relatively high. The best inhibitory efficiency showed the compound **T106** (Table 4). However, **T106** did not show any inhibitory efficiency (similar to compounds **T123** and **T125**) in *in vitro* experiments on model epithelial cell lines infected with SARS-CoV-2. Possible reasons for these findings, which may include, for example, different cellular permeabilities of the compounds or a different mechanism of action, are discussed in the following.

### Molecular modelling of PL^pro^ inhibition

Computational predictions of enzyme-inhibitor interaction energies ($\Delta E_{\text{int}},$ in kcal/mol) of the studied bis(benzylidene)cyclohexanone inhibitors and PL^pro^ from SARS-CoV-2 (Table 1), which were based on the model crystal structure of the noncovalent complex PL^pro^-GRL0617, resulted in the following order of calculated $\Delta E_{\text{int}}$^3^ of the compounds to PL^pro^:

$$\begin{array}{c} T123(-22.4)>T122(-20.8)=T98(-20.8)>\text{GRL}0617(C9)(-15.1)>T105(-11.3)>T106(-10.0)>\\ >T97(-7.7)>T120(-2.1)>T125(-0.4)>T121(0.9)>T124(1.2) \end{array}$$

**Figure 4.**
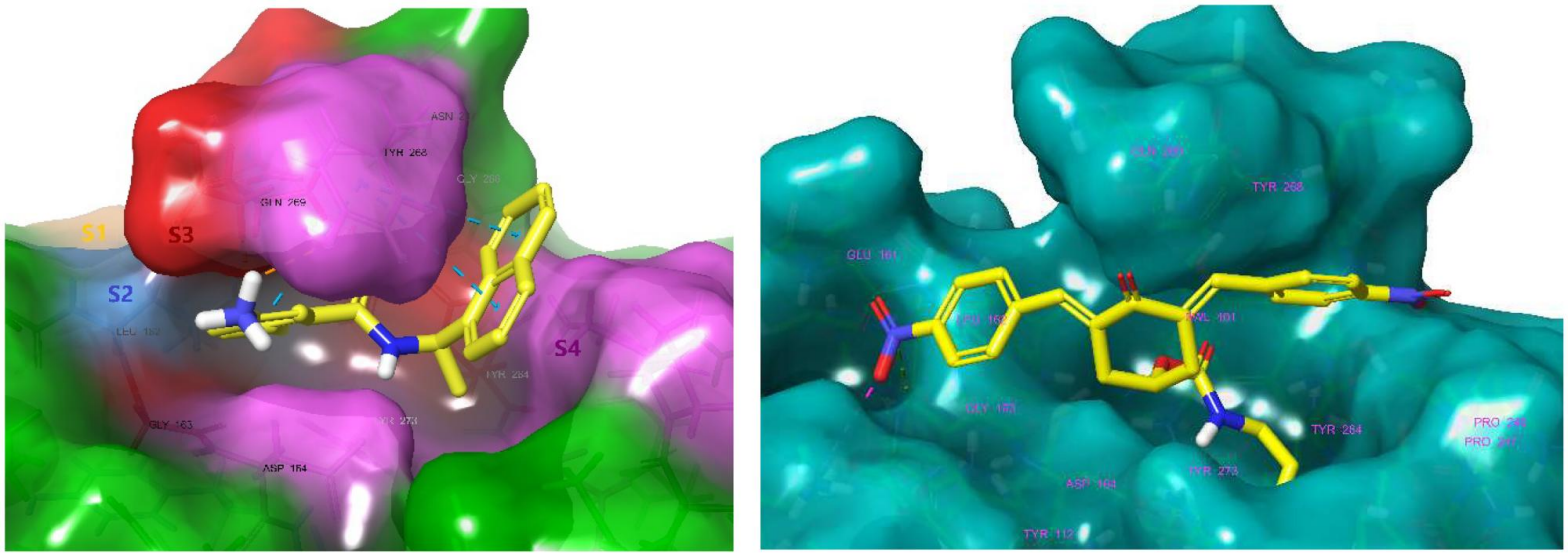
**Left:** Molecular surface of the binding site of GRL0617 that occupies the S_4_ - S_3_ pockets of the substrate binding cleft S_4_ - S_3_ - S_2_ - S_1_ of PL^pro^ of SARS-CoV-2. The flexible BL2 loop G266-NYQC-G271 stabilises the bound ligand in its position by closing on the inhibitor (PDB entry 7CMD) ^62^. **Right:** Binding site of the model of noncovalent complex PL^pro^ - **T98**. The partially transparent molecular surface of the enzyme illustrates the shape of the pockets of the substrate binding cleft. The amine tail is oriented downward and is not fully visible. Atom colouring scheme: H, white; C, yellow; N, blue; O, red. Only polar hydrogens are shown.

In the PL^pro^-inhibitor complexes of compounds **T97** - **T125**, the head group occupied similar positions in the S_4_ - S_3_ binding pockets, while most of the tail chains (substituents in the *4*-position of the cyclohexanone) were directed towards the catalytic site (S_2_ - S_1_). As expected, PL^pro^ with proven DUB activity recognises and preferentially binds thin aliphatic chains ending with a protonated amine group that mimics the side chain of lysine (as in **T123**, **T122**, and **T98**). These three compounds are predicted to form more stable associates with PL^pro^ (Figure 1) than the reference inhibitor GRL0617 (IC_50_ = 1.37 − 2.10 µM)^46^^,^^48^^,^^62^, Figure 4. The calculated $\Delta E_{\text{int}}$ are based on a non-covalent attachment of inhibitors to PL^pro^ disregarding possible subsequent nucleophilic addition of the thiol group of the catalytic Cys111. Therefore, it is not surprising that predicted $\Delta E_{\text{int}}$ of the compounds **T98** and **T122** are similar.

Irreversible inhibitors capable of forming a covalent bond with the catalytic cysteine of PL^pro^ will, according to our computational predictions, display inhibitory potencies related to their ability to undergo nucleophilic attack by the thiol group of Cys111 and consequently inactivate the enzyme, i.e., in the order of decreasing energy of the LUMO orbital of irreversible inhibitors. It was shown that within groups of structurally similar Michael acceptors, relative reactivities correlate with the LUMO energies^76^. Based on the DFT calculations, we can rank the BBC according to their LUMO energies (in a. u.):

$$\begin{array}{c} T122(-0.195)<T98(-0.191)<T123(-0.181)<\text{RA}-190(-0.157)<T124(-0.138)<T97(-0.098)<\\ <T121(-0.093)=\text{RA}-9(-0.093)<T106(-0.091)≅T120(-0.091)<T125(-0.069)<T105(-0.006) \end{array}$$

Compounds **T122** and **T125** were included in this array for comparison, despite not being Michael acceptors. According to LUMO orbital energies, the most reactive compounds **T98** and **T123** should be as potent or better irreversible inhibitors as the reference compound RA-190, Table 2. In general, molecular modelling suggests that compounds **T98**, and **T123** not only form stable initial noncovalent associates with PL^pro^ but should be potentially able to undergo nucleophilic addition of thiol group of cysteine to form a covalent complex with the PL^pro^, thus eventually lowering the catalytic efficiency of this enzyme.

On the other hand, the observed enzymatic inhibition by most BBC tested is relatively weak (IC_50_ values from 40 µM to >100 µM, Table 4), which suggests that PL^pro^ denaturation may have occurred rather than active site binding, as it was recently observed in high-throughput screenings of compound libraries for SARS-CoV-2 PL^pro^ inhibition^77^^,^^78^

### Concluding remarks

In summary, we evaluated the *in vitro* antiviral efficacy of 10 newly synthesised and 6 known inhibitors against SARS-CoV-2 using the cellular plaque reduction assay and explored the possible mechanism of the antiviral effect of the compounds by performing enzyme inhibition assays of the viral cysteine proteases M^pro^ and PL^pro^. Although *in vivo* animal models are preferred experimental systems for evaluating antiviral efficacy, *in vitro* testing using mammalian cells is a feasible option for assessing antiviral efficacy when animal models are not readily available. For that, cytotoxic activities were evaluated in various epithelial cell models such as VERO-E6, A549, PK-15, and Caco-2. This study was supplemented with evaluation of the enzymatic activities (deubiquitinase and protease activity) of the selected compounds. Gratifyingly, three compounds **T98**, **T120,** and **T121** showed a potent antiviral efficacy against SARS‐CoV‐2 (3.4 µM < EC_50_ < 8.4 µM) *in vitro* using VERO-E6 cells, while being tolerated by these cells (CTE > 10–15 µM). Furthermore, two of these three compounds (**T98** and **T120**) also exhibited a direct inhibitory effect towards the deubiquitinase and protease activities of PL^pro^ measured by enzyme assays (IC_50_), however, approximately 10 times weaker than the measured EC_50_ in the viral assay. Two more compounds **T122** and **T124** showed acceptable inhibitory potency against SARS-CoV-2 (EC_50_ of 17.0 and 15.9 µM, respectively) *in vitro* using the same cells, while being very well tolerated by these cells (CTE > 30 µM). One of these two compounds (**T122**) exhibited a direct inhibitory effect towards both the deubiquitinase and protease activities of PL^pro^ measured by enzyme assays (IC_50_), although approximately 4 times weaker than the measured EC_50_ in the viral assay.

The observed inhibitory activities (IC_50_ and EC_50_) towards SARS-CoV-2 do not correlate well with molecular modelling predictions of binding energies ($\Delta E_{\text{int}}$) and reactivities (q_C*_ and E_LUMO_) related to the multistep nucleophilic addition of the catalytic cysteine thiol group. The possible reason is, that molecular models assumed the binding mode of the bis(benzylidene)cyclohexanone headgroup to PL^pro^ similar to the noncovalent inhibitor GRL0617^62^. Based on the molecular models and calculated binding energies $\Delta E_{\text{int}}$ were able to identify analogs **T98**, **T106**, **T122**, and **T123**, which then showed the highest inhibitory effect towards the protease activity of PL^pro^ (IC_50_ = 40 – 60 µM) in the group of studied compounds, but $\Delta E_{\text{int}}$ could not reproduce the relative order of observed activity. Thus, we cannot conclude that BBC fully share the mode of action of GRL0617, which is a potent inhibitor of PL^pro^ (IC_50_ = 3.2 µM)^62^. Likewise, the order of LUMO orbital energies does not correlate well with the observed IC_50_. Therefore, nucleophilic addition is most probably not crucial for the mode of inhibition of PL^pro^ by the compounds studied. Obviously, the mechanism of action of BBC appears to be more complex and different from that considered in molecular modelling predictions, since enzyme assays showed only weak inhibitory potencies of **T98** – **T125** towards viral proteases M^pro^ and PL^pro^. Known irreversible deubiquitinase inhibitors RA-9 and RA-190 that share the structure of bis(benzylidene)cyclohexanone with the compounds tested also did not show an inhibitory effect on M^pro^ and PL^pro^ (IC_50_ > 100 µM). In a drug repurposing study^46^, Cho *et al.* experimentally screened libraries of deubiquitinase and cysteine protease inhibitors containing BBC on their inhibition of PL^pro^. They found that only one of them NSC63283 showed poor deubiquitinase activity (IC_50_ > 100 µM). It is, in fact, well known that Michael acceptors of this class can react with a variety of biological thiols^30^. Comparable inhibitory potencies *in vitro* of compounds **T98** (EC_50_ = 6.7 µM) and **T122** (EC_50_ = 17.0 µM) suggest that the reaction of the 1,5-diaryl-3-oxo-1,4-pentadienyl motif with the catalytic Cys111 thiol group is not essential for the observed antiviral effect in virus-infected VERO-E6 cells, as **T122** is not a Michael acceptor. Within the homologous series **T97**, **T98**, **T105**, **T106**, **T120**, **T121**, and **T124**, which differ only in substituent in the *4*-position of cyclohexanone, the antiviral effectiveness ranges from EC_50_ = 3.4 µM to EC_50_ > 30 µM, while inhibition of the cysteine protease activity of PL^pro^ and M^pro^ is weak (IC_50_ > 100 µM, except for **T106**). Therefore, we must conclude that, in analogy to RA-9 and RA-190, antiviral activity is mainly due to interactions with other targets^35^. The reference inhibitors included in our study PR-619, GRL0617, GC376 showed in our experiment inhibitory potencies IC_50_ corresponding to the data published in the literature^46^^,^^48^^,^^50^^,^^62^^,^^73^^,^^75^. Therefore, it could be hypothesised that the *4*-substituent group plays a specific role in inhibiting viral replication in cells by affecting replication pathways or pharmacological targets other than cysteine proteases. The important role of the *4*-substituent is also supported by the similar antiviral activity of **T122** vs. **T98** and **T97** vs. **T125,** but also by the loss of inhibitory activity (EC_50_ > 30 µM) when the *4*-substituent group becomes too bulky (**T105**) or too long (**T106**). Compound **T122** has moderate antiviral and inhibitory activities while **T125** shows only moderate inhibitory activity and no antiviral activity. This reinforces the conclusion that there is no relationship between *in vitro* antiviral activity and the PL^pro^ and M^pro^ activity of these compounds.

Although potent inhibition in an enzyme assay cannot guarantee antiviral activity in cell-based assays (nor in animal studies), we accessed both evaluations to determine antiviral efficiency of the new compounds and explore their mechanism of action. Measured inhibitory potencies (EC_50_ < IC_50_) were thus influenced by a complex interplay of multiple factors in the cellular environment, which includes pharmacological target(s) other than PL^pro^ and M^pro^, metabolic activation of the tested compounds, target location, structural complexity of cells, transport mechanisms, and others. Therefore, it is important to measure both IC_50_ and EC_50_ values to fully understand the pharmacological profile of a novel compound, as they provide complementary information about the compound’s potency and efficacy.

It is noteworthy that all our active compounds exhibited effective *in vitro* inhibitory concentrations EC_50_ comparable to those of commercially available inhibitors having similar chemical structures (e.g., PR-619, RA-9, RA-190) as well as to those of approved FDA drugs (e.g., remdesivir **C8**). In general, our study has identified new chemical entities, antiviral compounds as possible candidates for further *in vivo* testing and for eventual advancement to preclinical development.

## Supplementary Material

Supplemental Material

## Notes

1 Interferon-stimulated gene 15 (ISG15) is a secreted protein that covalently links to lysine residues on newly synthesized proteins. The effects of protein ISGylation involve activation and inhibition of antiviral immunity.

2 LUMO - lowest unoccupied molecular orbital.

3 More negative value of $\Delta E_{\text{int}}$ means stronger binding of the ligand to PL^pro^.
