# Supplementary material for: Identification and evaluation of antiviral activity of novel compounds targeting SARS-CoV-2 virus by enzymatic and antiviral assays, and computational analysis
Source: J Enzyme Inhib Med Chem. 2024 Jan 14;39(1):2301772. doi: 10.1080/14756366.2024.2301772 (PMC10791089; doi:10.1080/14756366.2024.2301772)
Supplement: Supplemental Material [file IENZ_A_2301772_SM5731.docx]

**Supplementary Material**

**Identification and evaluation of antiviral activity of novel compounds targeting cysteine proteases of SARS-CoV-2 by enzymatic and antiviral assays, and computational analysis**


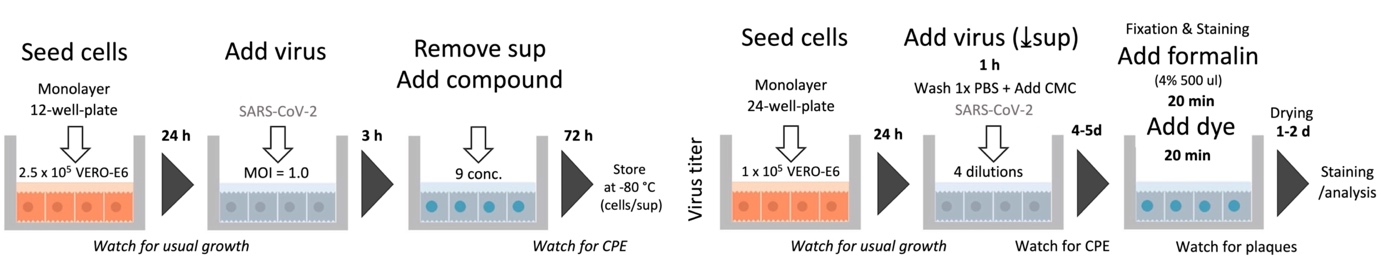


**Figure S1.**

Scheme of the biological evaluation of the compounds studied. The assay was performed *in vitro* in the presence of a live SARS-CoV-2 virus. Details of the format of the test plates, time intervals, and working steps are shown here, as well as mentioned in the Materials and Methods section of the paper.


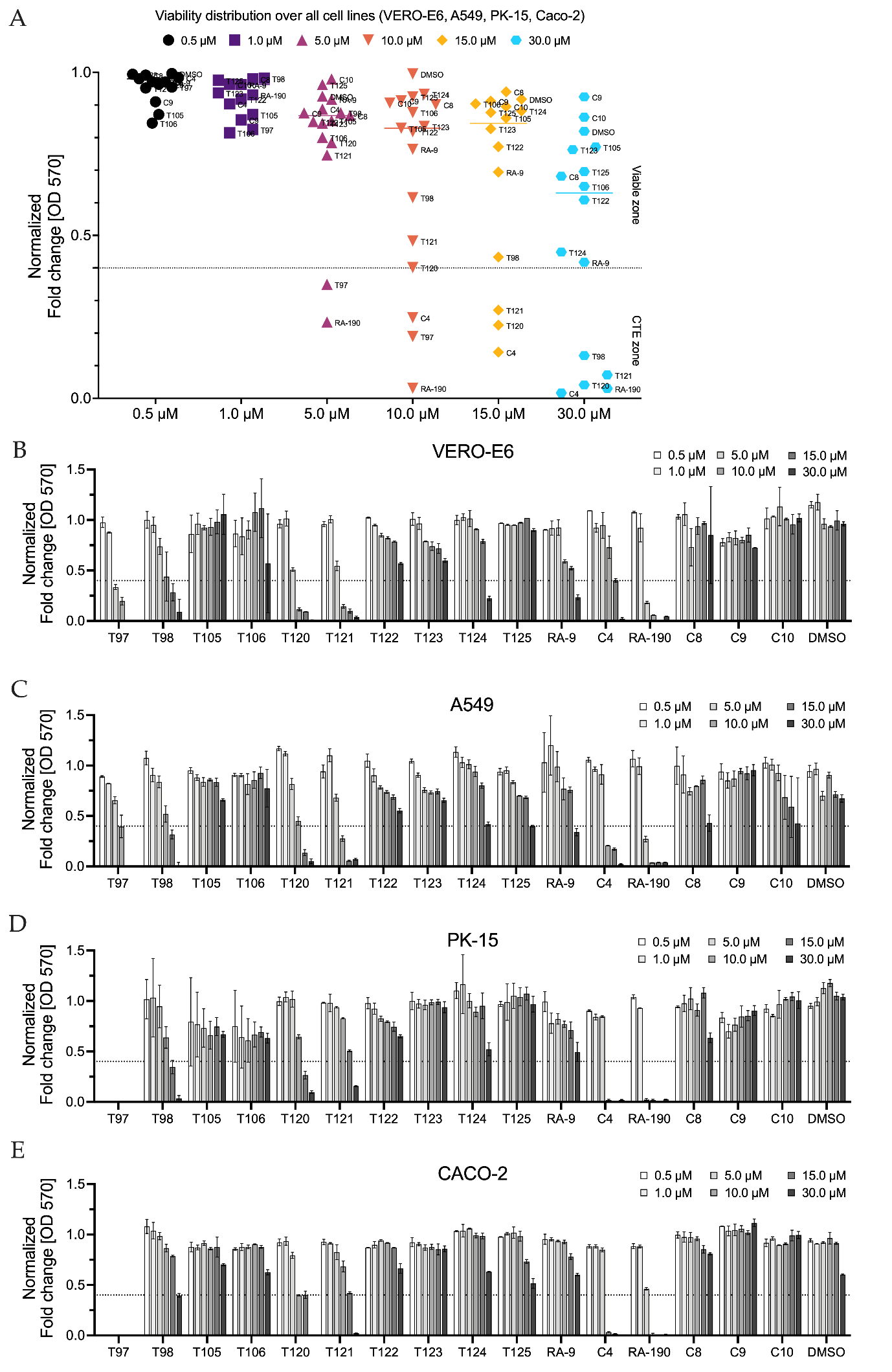


**Figure S2.**

Apoptotic responses measured spectrophotometrically by OD at 570 nm (formazan absorption) induced by the compounds studied in VERO-E6 (**B**), A549 (**C**), PK-15 (**D**), Caco-2 cells (**E**) and averaged in all cell lines (**A**). Cells were treated with the indicated concentrations of different testing (**T97-T98**, **T105, T106**, **T120-T125, RA-9, RA-190**) and reference compounds (**C4**, **C8-C10**) for 72 hours. Cell viability as visualized by fold change of formazan-positive cells normalized to the untreated control. Data are presented as mean with SD. The results could be compared with cells treated with DMSO (at the end of the plots B-E). Viability reduced below 40% represents the cytotoxic zone (CTE zone), below the dotted line in each plot (A-E). Compound **T97** was tested on VERO-E6 and A549 only.

**
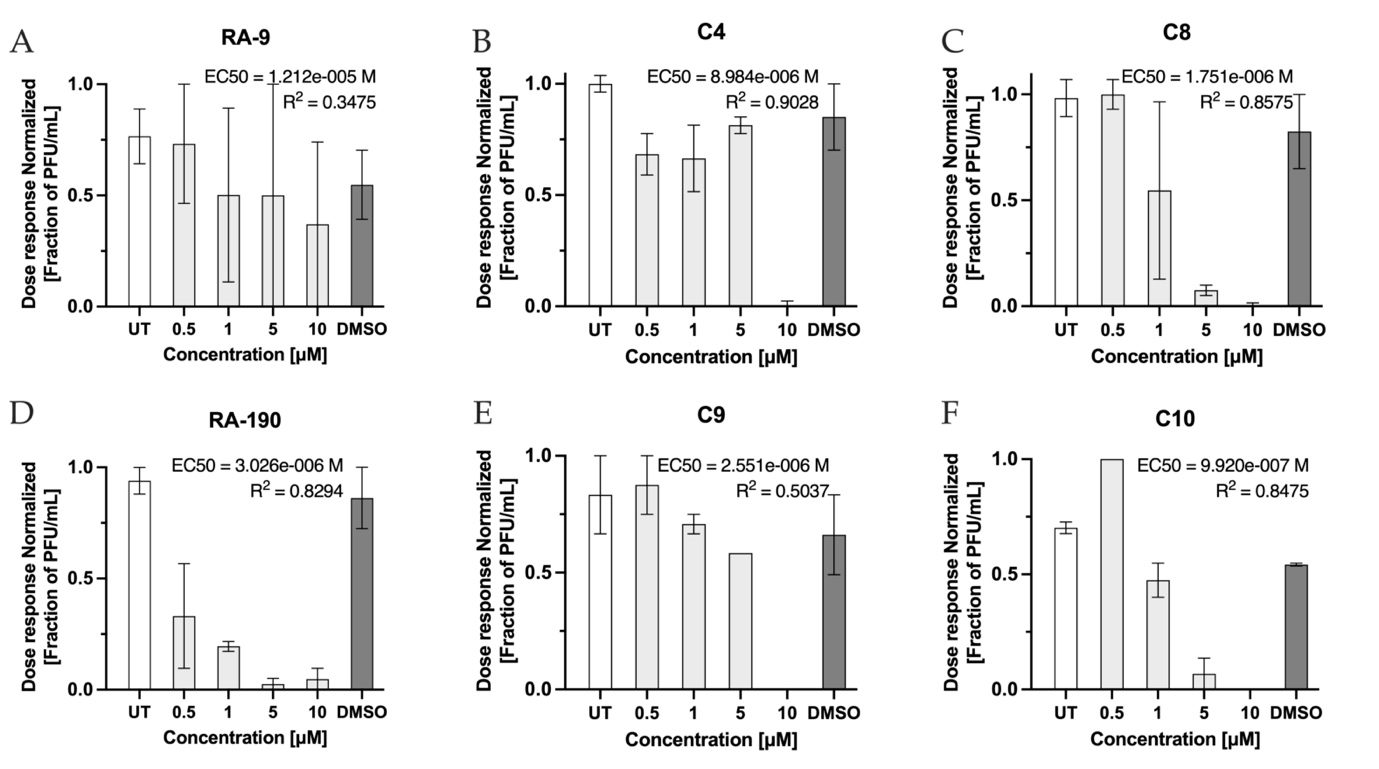
**

**Figure S3.**

Evaluation of antiviral efficacy with calculation of the effective inhibitory concentration EC_50_ shown for reference inhibitors: (**A**) RA-9; (**B**) PR-619 (**C4**); (**C**) remdesivir (**C8**); (**D**) RA-190; (**E**) GRL0617 (**C9**); and (**F**) GC376 (**C10**). Monitoring the trend of decreasing the titer of the SARS-CoV-2 virus (normalized to an untreated control) with increasing compound concentration (A-F). Data are presented as mean with SEM. Determination of the half-maximal effective concentrations EC_50_ [M] from the sigmoidal model with the corresponding coefficient of the regression determination represented by R^2^ (the goodness of fit). For simplicity, the sigmoidal model is not shown here, the EC_50_ [M] and R^2^ values are shown in the plot.
